# Exploring the use of cluster analysis to assess antibiotic stewardship in critically-ill neonates in a low resource setting

**DOI:** 10.1186/s13756-023-01325-w

**Published:** 2023-10-31

**Authors:** Roberto Benoni, Eleonora Balestri, Tariqua Endrias, Jiksa Tolera, Martina Borellini, Margherita Calia, Filippo Biasci, Luigi Pisani

**Affiliations:** 1 Department of Woman’s and Child’s Health, University of Padua, Via Giustiniani, 3, Padua, 35128 Italy; 2 Section of Hygiene, Department of Diagnostics and Public Health, University of Verona, Strada Le Grazie, 8, Verona, 37134 Italy; 3 Section of operational research, Doctors with Africa CUAMM, Padova, Italy; 4 Neonatal Intensive Care Unit, AUSL-IRCCS of Reggio Emilia, Reggio Emilia, Italy; 5 Doctors with Africa CUAMM Ethiopia, Wolisso, Ethiopia; 6 Neonatal Intensive Care Unit, St Luke Catholic Hospital, Wolisso, Ethiopia; 7 Mahidol Oxford Research Unit, Bangkok, Thailand

**Keywords:** Neonatal sepsis, Antibiotic stewardship, Cluster analysis, Multidrug resistance

## Abstract

**Background:**

Sepsis is the third leading cause of neonatal death in low and middle-income countries, accounting for one third of all deaths in Ethiopia. A concerning issue is the increasing number of multidrug-resistant microorganisms facilitated by suboptimal antibiotic stewardship. The study aims to identify clusters of newborns switching antibiotic lines for sepsis in a neonatal intensive care unit (NICU) in Ethiopia, and to explore their potential association with sepsis outcomes.

**Methods:**

A retrospective cohort study was conducted including all newborns discharged with a diagnosis of probable neonatal sepsis from the St. Luke Catholic Hospital NICU between April and July 2021. The antibiotic management protocol included two lines according to WHO guidelines and a third line based on internal hospital guidelines. In the cluster analysis, the Gower distance was estimated based on the antibiotics employed in the different lines and the duration of each line. Mortality and respiratory distress (RD) were the response variables.

**Results:**

In the study period, 456 newborns were admitted to the NICU and 196 (42.8%) had probable neonatal sepsis. Four antibiotic management clusters were identified. Cluster 1 (n = 145, 74.4%) had no antibiotic switches, using only the first line. Cluster 2 (n = 26, 13.3%) had one switch from the first to the second line. Cluster 4 (n = 9, 4.6%) had two switches: from first to second and then to third line. In cluster 3 (n = 15, 7.7%), newborns were switched from ceftriaxone/cloxacillin as second line to off-protocol antibiotics. There were no differences in sex, age, weight on admission or crude mortality between clusters. Cluster 3 included a higher frequency of infants who did not breathe at birth (53.3%, p = 0.011) and that necessitated bag ventilation (46.7%, p = 0.039) compared to the other clusters.

**Conclusions:**

The first antibiotic line failed in one out of four newborns with probable sepsis while third-generation cephalosporins were insufficient in one in ten patients. Cluster analysis can provide valuable insights into antibiotic treatment patterns and their potential implications. This approach may support antibiotic stewardship and aid in contrasting antimicrobial resistance in limited resource settings.

**Supplementary Information:**

The online version contains supplementary material available at 10.1186/s13756-023-01325-w.

## Background

Neonatal mortality is a concerning issue in low- and middle-income countries (LMIC) where most of the estimated 2.6 million yearly global deaths occur. Neonatal sepsis is the third leading cause of neonatal mortality being responsible for 13% of all neonatal deaths, and of 42% of deaths in the first week of life [1]. In Ethiopia, prematurity (37%), sepsis (28%), and asphyxia (24%) are reported to be the most common causes of death in newborns [2].

Neonatal sepsis is defined by the systemic manifestation of infection, due to the presence in the bloodstream of a bacterial pathogen. Neonatal sepsis is classified as ‘confirmed’ if bacteremia is proved by a positive blood culture, ‘probable’ if signs and symptoms are supported by suggestive laboratory results, and ‘suspected’ if only clinical suspicion is present [1]. Differentiating between early-onset sepsis (EOS) and late-onset neonatal sepsis (LOS) is important for tailored management of etiological pathogens [1–3]. Respiratory distress (RD) is one of the most common severe clinical presentations of neonatal sepsis and it is associated with poor outcomes [4, 5].

Multidrug resistance for pathogens involved in sepsis is worsening, both globally and in LMICs, with greater attention advocated for antibiotic stewardship [6]. Antimicrobial resistance, or reduced susceptibility, to the combination of penicillin and gentamicin and to third-generation cephalosporins was reported, in more than 40% of cases of neonatal bacteremia acquired in a community setting [7]. Overall resistance to third-generation cephalosporins in Gram-negative bacteria, estimated to be around 50% in Africa, is increasing [8]. Recent initiatives advocate the need to investigate new management algorithms to reduce unnecessary antibiotic use for neonatal sepsis, especially in the neonatal intensive care unit (NICU) setting [9].

Cluster analysis is a machine learning technique that aims to group observations that are similar within the same cluster. This analysis includes a cluster construction phase and a subsequent validation phase. There may be an internal validation (calculating an index to assess how well the clusters fit the data) and an external validation (using an external dataset or survival curves) [10]. The development of decision support systems based on historical data using machine learning algorithms is an opportunity in healthcare to obtain predictive information and improve decision making and clinical practice [11]. This can be particularly useful in resource-limited settings. Cluster analysis was already applied in infectious disease, analyzing biomarkers and predictors of neonatal sepsis, but to the best of our knowledge, no study has applied it to antibiotic stewardship in LMICs NICU setting [12, 13]. Therefore, we aimed to identify clusters of patients switching between different antibiotic lines and to explore their potential associations with survival probability or severe clinical presentation in terms of RD. We also sought to report the prevalence of early versus late probable neonatal sepsis in this setting. The primary hypothesis is that specific clusters of antibiotic switching can be identified, and that they are associated with more severe clinical presentation and poorer outcome.

## Methods

### Study Design

We used a retrospective cohort study design to explore the use of cluster analysis in depicting antibiotic management of neonatal sepsis and its association with newborns’ outcomes in a limited resources NICU.

### Ethical approval

The research was performed following the ethical standards of the 1964 Declaration of Helsinki and was approved by the Ethical Committee of St. Luke Catholic Hospital (SLCH) on the 14th of September (protocol number 1293/2021).

### Study setting

SLCH is a referral hospital located in Wolisso Town, about one hundred km from the capital Addis Ababa. It is situated in the South-West Shoa Zone (SWSZ) of the Oromia region (Ethiopia), with an estimated population of 1,311,406 inhabitants, of which 15% are under five years of age [14]. SLCH catchment area includes the *woreda* of Ameya, Wenchi, Waliso rural, Woliso town, Becho, and Goro representing the reference hospital for 743.797 individuals. The number of deliveries assisted at SLCH was 4455 in 2019 and 4015 in 2020. The SLCH NICU has 16 beds with an annual average bed-occupation rate of 112% in 2020 [15]. Vital parameters and blood oxygen saturation are routinely monitored, and respiratory support comprises intranasal oxygen, bubble CPAP and electric CPAP.

### Population

All newborns admitted in NICU between 1st April 2021, and 31st July 2021 and discharged with the diagnosis of probable neonatal sepsis were included. Exclusion criteria were missing information about antibiotic management or regarding the type of neonatal sepsis.

### Operational definitions

Probable neonatal sepsis was defined as the presence of two or more of the following clinical signs and symptoms: hypo-hyperthermia (BT < 35.5 °C or > 37.5 °C), heart rate > 180 or < 100 bpm, respiratory rate > 60 breaths/min with grunting or desaturations, lethargy or altered mental status, glucose intolerance (plasma glucose > 10mmol/l), feed intolerance; plus at least one of the following laboratory results at birth: leukocytosis (WBC count > 34,000*109/l), leukopenia (WBC count < 5,000*109/l), thrombocytopenia (platelets count < 100,000*109/l) [1].

Neonatal sepsis was defined as early-onset (EOS) if the sepsis symptoms started within 72 h of birth and late-onset (LOS) if they began later than72 hours from birth [2, 3].

Respiratory distress was defined as the presence of two or more of the following signs: abnormal respiratory rate (> 60 or < 30 breaths/minute, respiratory pauses, or apnea), grunting, nasal flaring, intercostal recessions, xiphoid recessions, with or without cyanosis [16]. RD was assessed at the time of admission to the neonatal intensive care unit and daily throughout the entire length of stay.

### Data collection

For each subject, the following data were collected: sex, age and weight at admission, date of hospital admission and of hospital discharge, number of antenatal care (ANC) visits, mode of delivery, delivery place, presence of maternal chorioamnionitis, occurrence of premature rupture of membranes (PROM), maternal pre-eclampsia/eclampsia, Apgar score at 1st, 5th, 10th minute, respiratory status at birth, use of oxygen and/or positive pressure ventilation with AMBU bag at birth. We also collected data on type of neonatal sepsis, type of every antibiotic used during hospitalization, start and end date of every antibiotic type, presence of RD, number, type and length of every respiratory support device used during hospitalization and the outcome at NICU discharge. Due to the study setting (a low-resource neonatal intensive care unit), microbiological tests (pathogen typing and AMR profiling) were not available and were therefore not included in the analysis.

### Antibiotic regimens

The antibiotic management protocol at the SLCH NICU had three different antibiotic lines. The first two lines were based on WHO and Ethiopian guidelines [17]: the first line was ampicillin plus gentamicin and the second line included ampicillin (higher dosage) – or cloxacilline if any sign or suspicion of staphylococci infection, plus cefotaxime or ceftazidime as the first choice - or ceftriaxone if the previous two were not available. The third line used ciprofloxacin plus cloxacilline (or vancomycin). Ciprofloxacin was empirically chosen as a third line as in Addis Abeba Central Hospital, where blood cultures and antibiotic resistance profile are available, several cases of resistance to gentamicin and cephalosporins but high sensitivity to meropenem and ciprofloxacin were found [18]. First line duration of antibiotic treatment was recommended for 5–7 days. If no improvement was observed in the first 48 to 72 h, the second line regimen was started. The third line was under specialist prescription based on the clinical status of the newborns.

### Study endpoints

The primary endpoints were the number of newborns assigned to each cluster of antibiotic line switch and the development of respiratory distress or death in NICU. The secondary endpoints were the proportion of patients diagnosed with early and late-onset neonatal sepsis subtypes.

### Statistical analysis

For descriptive purposes, frequency rates and percentages were used for categorical variables and medians with interquartile range (IQR) for continuous variables. Proportions for categorical variables were compared by the χ2 and Fisher’s exact test. Continuous variables were compared via Mann-Whitney-U non-parametric test.

The optimal number of antibiotic management clusters to be imputed in the algorithm was evaluated through silhouette coefficient fitted on Gower distance computed as the average of partial dissimilarities across individuals included in the study. Variables included to be used in the Gower distance estimation were the type of antibiotics used as the first, second, or third line, respectively, and the length of therapy (LOT, in days) of each antibiotic line. The Gower distance was selected because data had both continuous and categorical variables, and it allows for mixed variables to be used simultaneously. The individuals were assigned to the different clusters through the partitioning around medoids (PAM) technique with the k-medoids algorithm fitted on the previous computed optimal number of clusters and Gower distance [19].

The PAM algorithm is based on the search for medoids (‘k’ representative objects) among the observations of the database. It has two phases. Build phase: (1) select k objects that will become the medoids; (2) calculate the dissimilarity matrix; (3) assign each object to the nearest medoid. Swap phase: (4) for each cluster, assess whether one of the objects decreases the average dissimilarity coefficient; if so, this is selected as the new medoid for this cluster; (5) if at least one medoid has swapped, go to step 3, otherwise end the algorithm. The goal of the algorithm is to minimize the average dissimilarity of objects with respect to the closest selected object.

The differences between the clusters in terms of descriptive socio-demographic and clinical characteristics were assessed via Fisher’s exact test and the non-parametric Mann-Whitney-U test for categorical and continuous variables, respectively.

The median survival time was examined by Kaplan–Meier estimates. To assess the effect of the antibiotic management clusters and the clinical variables on mortality a Cox Proportional-Hazards model was used with type of neonatal sepsis, age, weight, antibiotic management cluster, and presence of RD as potential determinants. Results were presented as hazard ratio (HR) with 0.95 confidential interval (CI). Since time at RD onset was not available, to explore its association with type of neonatal sepsis, age, weight, and antibiotic management clusters a logistic regression model was applied. Results were presented as odds ratio (OR) with 0.95CI. Additionally, the main clinical features were tested between clusters through Fisher’s exact test and Mann-Whitney-U non-parametric test. Dunn’s test with Bonferroni adjustment was used for nonparametric pairwise multiple comparisons. Post-hoc multiple comparison between clusters in the logistic regressions was carried out through Tukeys’ test.

Finally, the same set of analyses (χ2, Fisher’s exact test and non-parametric Mann-Whitney-U test, logistic regression) was conducted to assess differences in the distribution of the main clinical characteristics between patients with EOS and LOS.A p-value < 0.05 was considered significant. All analyses were performed using the R software (version 4.1.1) using package “cluster”, “ggplot2” and “Rtsne” to perform the cluster analysis and data visualization [20].

## Results

### Patients’ characteristics

**Fig. 1:**
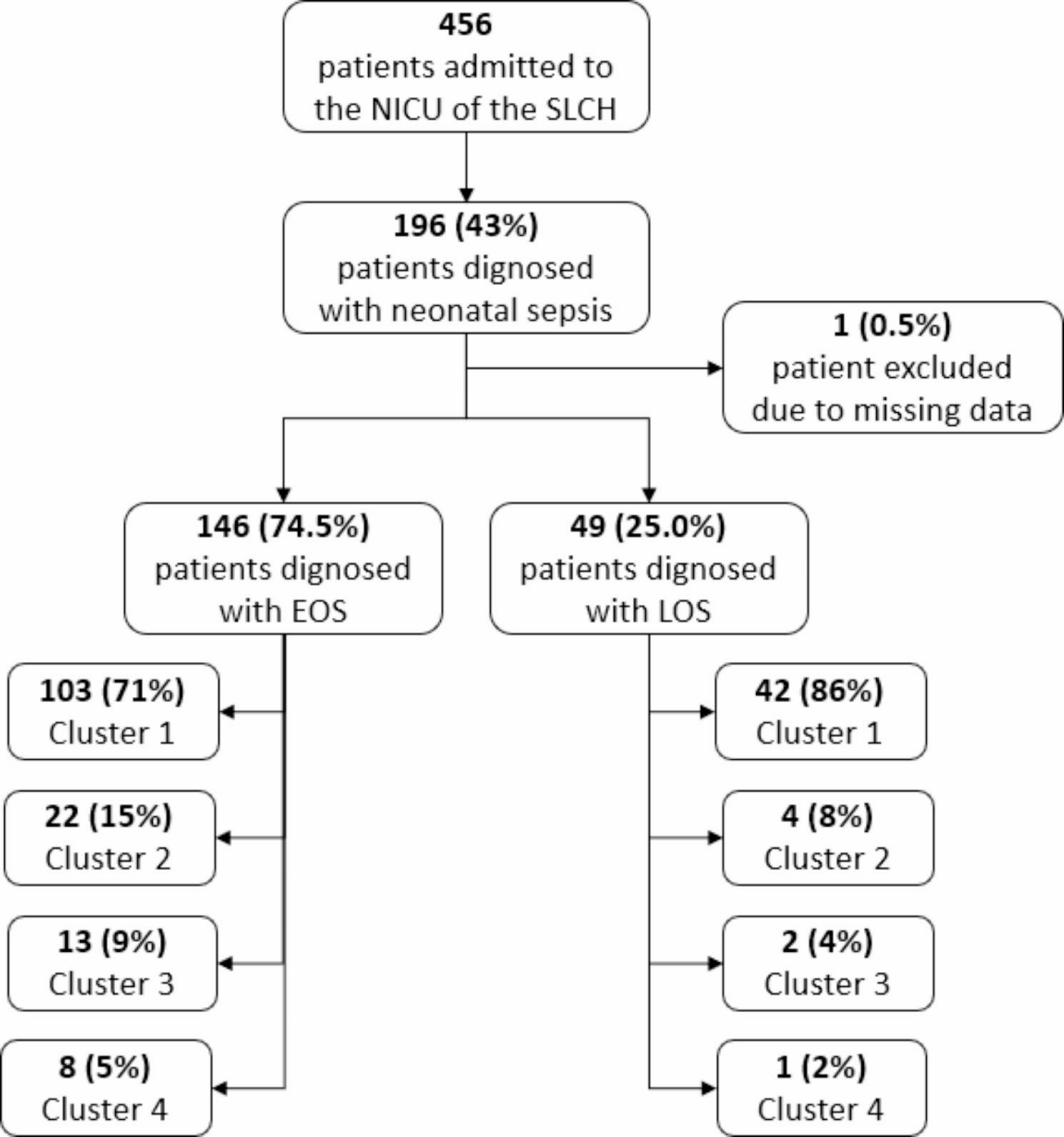
Flowchart of the newborns sample included in the study. EOS = Early-Onset Neonatal Sepsis; LOS = Late-Onset Neonatal Sepsis; NICU = Neonatal Intensive Care Unit; SLCH = St. Luke Chatolic Hospital (Ethiopia

In the study period 456 newborns were admitted to the NICU of SLCH and 196 (42.8%) were discharged with a diagnosis of probable neonatal sepsis. One newborn was excluded due to missing data (Fig. 1). EOS was the predominant phenotype with 146/195 newborns (74.9%). Median age and weight at admission were 1.0 day (IQR 1.0-5.5) and 2,900 g (IQR 2,315-3,300), respectively. The majority of neonates were hospital inborn (119–61.0%). The prevalent mode of delivery was spontaneous vaginal delivery followed by C- section and assisted breech (Table 1).

**Table 1:** Demographic, epidemiological, and clinical characteristics of newborns with probable neonatal sepsis, distinguished by the type of neonatal sepsis

	Overall (n = 195)	EOS (n = 146)	LOS (n = 49)	p-Value*	
**Sex**				0.319	
Female	83 (42.6%)	59 (40.4%)	24 (49.0%)		
Male	112 (57.4%)	87 (59.6%)	25 (51.0%)		
**Age (day)**				< 0.001	
Median (IQR)	1.0 (1.0-5.5)	1.0 (1.0–2.0)	15.0 (10.0–21.0)		
**Weight at admission (g)**				< 0.001	
Median (IQR)	2900 (2315–3300)	2710 (2100–3200)	3100 (2725–3690)		
**Place of delivery**				0.008	
Inborn	119 (61.0%)	102 (69.9%)	17 (34.7%)		
Health center	49 (25.1%)	32 (21.9%)	17 (34.7%)		
Home	27 (13.8%)	12 (8.2%)	15 (30.6%)		
**Mode of delivery**				0.002	
Spontaneous vaginal delivery	136 (69.7%)	92 (63.0%)	44 (89.8%)		
C- section	38 (19.5%)	33 (22.6%)	5 (10.2%)		
Vacuum	15 (7.7%)	15 (10.3%)	0 (0.0%)		
Assisted breech	6 (3.1%)	6 (4.1%)	0 (0.0%)		
**Premature rupture of membranes (N/A = 2)**				0.004	
	41 (21.0%)	38 (26.0%)	3 (6.1%)		
**Chorioamnionitis (N/A = 2)**				0.015	
	19 (9.7%)	19 (13.0%)	0 (0.0%)		
**Eclampsia (N/A = 2)**				0.999	
	3 (1.5%)	3 (2.1%)	0 (0.0%)		
**Apgar 1st minute**				0.109	
Median (IQR)	6.0 (3.0–8.0)	6.0 (3.0–7.0)	8.0 (3.5-9.0)		
**Apgar 5th minute**				0.081	
Median (IQR)	7.0 (6.0–9.0)	7.0 (6.0–9.0)	9.0 (4.5–10.0)		
**Breath at birth (N/A = 2)**				< 0.001	
	155 (79.5%)	108 (74.0%)	47 (95.9%)		
**Positive pressure ventilation at birth (N/A = 2)**				< 0.001	
	39 (20.0%)	39 (26.7%)	0 (0.0%)		
**Oxygen given at birth (N/A = 2)**				< 0.001	
	47 (24.1%)	47 (32.2%)	0 (0.0%)		
**Respiratory distress syndrome**				0.412	
	90 (46.2%)	70 (47.9%)	20 (40.8%)		
**Length of stay**				0.119	
Median (IQR)	7.0 (6.0–11.0)	8.0 (6.0–11.0)	7.0 (5.0–9.0)		
**Outcome at discharge**				0.014	
Alive	161 (82.6%)	114 (78.1%)	47 (95.9%)		
Dead	26 (13.3%)	24 (16.4%)	2 (4.1%)		
Referred	8 (4.1%)	8 (5.5%)	0 (0.0%)		

* Fisherman-s exact and χ2 test, Mann-Whitney-U non-parametric test.

** Early (EOS) and late (LOS) neonatal sepsis.

N/A = data were missing in the medical records.

### Antibiotic management clusters

**Fig. 2:**
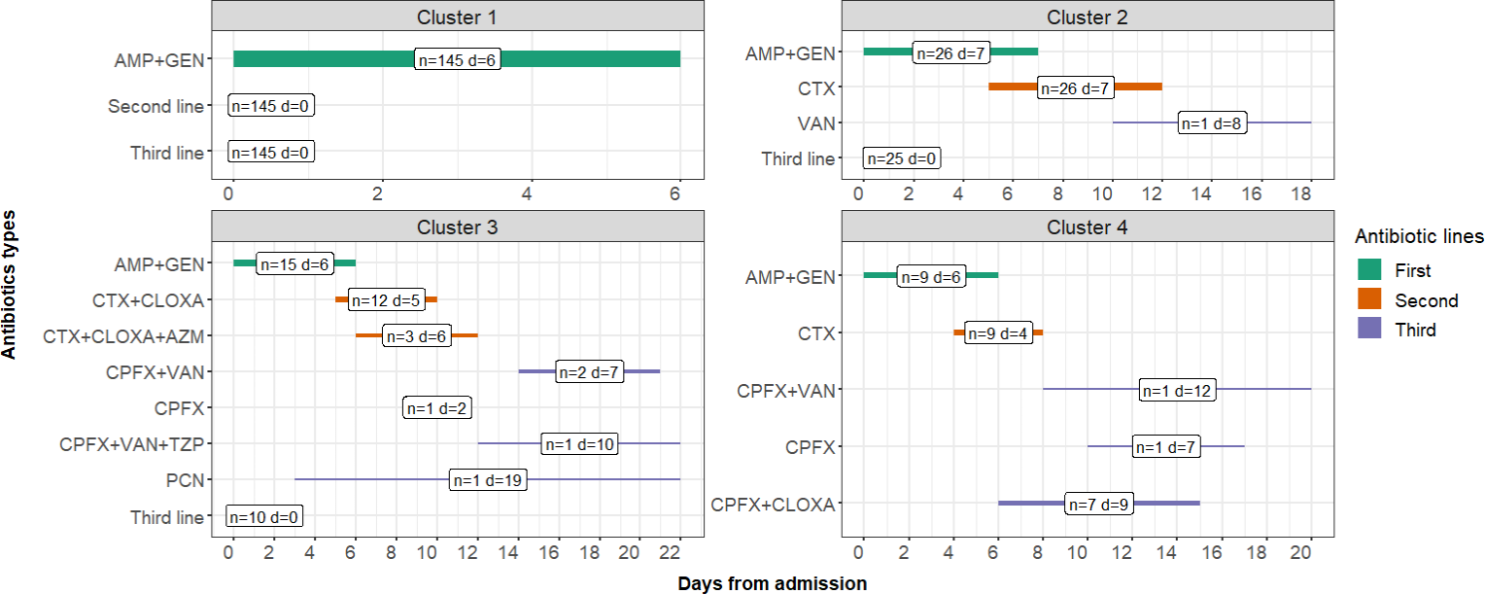
Graphical representation of the 4 identified neonatal sepsis antibiotic management clusters. The bars represent the duration in days of treatment of each antibiotic or combination of antibiotics. “n” is the number of subjects for each antibiotic line in the different clusters. “d” is the median duration in days of each type of antibiotic combination. AMP = Ampicillin, GEN = Gentamicin, CTX = Ceftriaxone, VAN = Vancomycin, CLOXA = Cloxacillin, AZM = Azithromycin, CPFX = Ciprofloxacin, TZP = Piperacillin /Tazobactam, PCN = Penicillin

All patients with neonatal sepsis received at least the first antibiotic line. The second and third line of antibiotic therapy were used in 50 (25.6%) and 15 (7.7%) neonatal sepsis cases, respectively. Four antibiotic management clusters were identified (**Table S2**, Fig. 2).

**Fig. 3:**
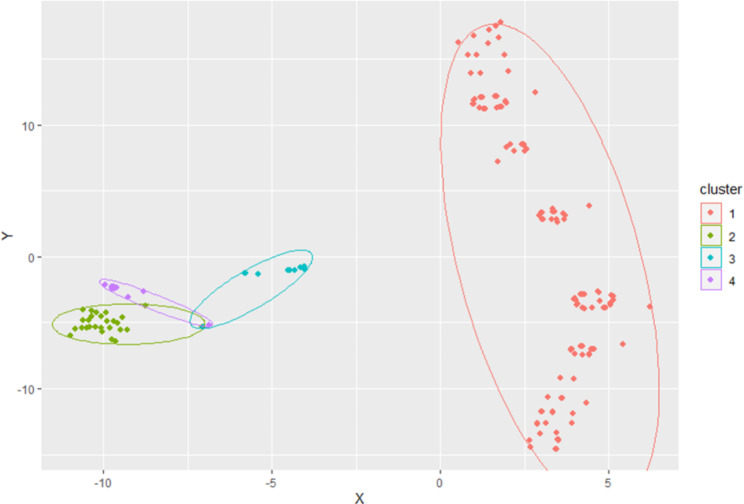
Scatter plot visualization of the four clusters of antibiotic management. The scatter plot used the t-distributed stochastic neighborhood embedding over the Gower distance estimated on type of antibiotics used as the first, second, or third line, respectively, and the length in days of each antibiotic line

The silhouette coefficient for a number of cluster k = 4 was 0.788, indicating a very good clustering (Fig. S1). Data visualization of the four clusters is provided in Fig. 3. The first cluster included 145 (74.4%) patients with no switching thus requiring only the first antibiotic line. In the second cluster there were 26 (13.3%) newborns switching from first line to ceftriaxone as second antibiotic line. The third cluster was made by 15 newborns who switch from the first line to a second line of ceftriaxone plus cloxacillin in 12 cases (6.2%), and plus a further second line antibiotic (azithromycin) in 3 cases (1.5%). A further switch to off-protocol antibiotics was required by 5 (2.7%) patients in cluster 3. The fourth cluster was made by 9 (4.6%) patients with two switches: from first to second line (ceftriaxone only) and from second to the third antibiotic line (ciprofloxacin plus cloxacillin) in 7 (3.6%) cases.

Demographic and clinical characteristics stratified by cluster are shown in Table S1. There were no differences in sex (p = 0.693), admission weight (p = 0.432), type of sepsis (p = 0.274), and Apgar at 1st (p = 0.430) and 5th (p = 0.264) minute between the antibiotic management clusters. Type of respiratory support and ventilation length were also not different between the clusters (p = 0.221). The frequency of newborns who did not breathe at birth (n = 8, 53.3%, p = 0.011) and required manual positive pressure ventilation (n = 7, 46.7%, p = 0.039, Table S1) was higher in cluster 3. Length of stay was shorter in cluster 1 when compared to the other clusters (p < 0.001, Table S1).

The probability of developing RD for newborns with neonatal sepsis was 91% and 87% higher for antibiotic management cluster 3 compared to clusters 1 and 2, respectively. The odds of developing RD decreased by 6.4% for the increment of 100 g in body weight at admission (p = 0.006) and was higher in the cluster 3 group when compared with clusters 1 and 2 (Table 2).

**Table 2:** Results of the logistic regression models fitted on the presence of respiratory distress (RD) as dependent variable and age, weight at admission, sepsis type, antibiotic management cluster as potential determinants. Cluster 3 was chosen as reference because it accounts for the cluster outside the protocol antibiotic lines

	Odds Ratio	0.95 CI	p-Value
**RD (yes/no)**			
Admission age (days)	1.027	0.930–1.135	0.592
Admission weight (hg)	0.936	0.893–0.980	0.006
Neonatal sepsis type (LOS)	0.855	0.184–3.811	0.838
Antibiotic cluster (3)			
1	0.088	0.013–0.357	0.003
2	0.133	0.018–0.637	0.022
4	0.389	0.037–3.981	0.405

*Early (EOS) and late (LOS) onset neonatal sepsis*

*Respiratory distress (RD)*

**Fig. 4:**
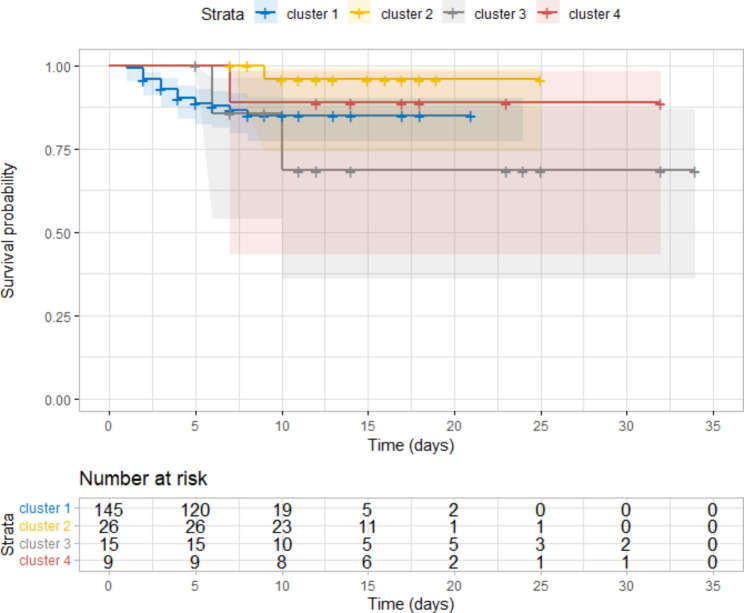
Kaplan–Meier curves for survival probability in newborn with neonatal sepsis distinguished by antibiotic management clusters

Since less than 50% of the cohort died (death rate = 13.3%), the Kaplan-Meier estimate median survival time was not computed (Fig. 4). Death HR was not significantly different based on assignment to antibiotic management clusters (Table 3). Death probability was 6.3 times higher for patients who developed RD (p = 0.004) compared to newborns who did not and decreased by 8% for the increment of 100 g in body weight at admission (p = 0.006).

**Table 3:** Survival hazard ratio (HR) estimated in the multivariable Cox Proportional-Hazards model with age, weight at admission, sepsis type, antibiotic management cluster, and the presence of RD as potential determinants. Cluster 3 was chosen as reference because it accounts for the cluster outside the protocol antibiotic lines

	HR	0.95 CI	p-Value
**Outcome (dead/alive)**			
Admission age (days)	1.13	0.85–1.50	0.414
Admission weight (hg)	0.92	0.86–1.50	0.006
Neonatal sepsis type (LOS)	0.04	0.00-7.66	0.235
RD	6.30	1.78–22.22	0.004
Antibiotic cluster (3)			
1	1.16	0.38–3.54	0.788
2	0.18	0.02–1.62	0.126
4	0.40	0.04–3.57	0.410

*Early (EOS) and late (LOS) onset neonatal sepsis*

*Respiratory distress (RD)*

### Clinical features of EOS and LOS

The main clinical features are listed in Table 1. EOS was associated with both PROM and chorioamnionitis (p = 0.004; p = 0.015); no differences were found in the frequency of mothers with pre- or eclampsia between the two types of neonatal sepsis (p = 0.998). RD was observed in 90 (46.2%) newborns with neonatal sepsis, 70 (47.9%) in EOS and 20 (40.8%) in LOS. Newborns requiring electric-CPAP were 30 (33.3%), bubble-CPAP were 41 (45.6%) and only intranasal oxygen was required by 19 (21.1%) patients. No differences were found between respiratory support types in the two groups of neonatal sepsis (p = 0.122). Risk of RD and death was not different between the two sepsis types groups (p = 0.838, p = 0.268, Table 3).

## Discussion

In this proof-of-concept study we explored the use of cluster analysis as a tool to discern the antibiotic lines used for neonatal sepsis in a resource-limited setting. Clustering allowed to identify a group of patients needing advanced or off-protocol antibiotic lines, and to assess frequency and types of switching between antibiotic lines in complex critically ill newborns. While three of the four clusters reflected the three lines of antibiotic therapy set out in the protocol (cluster 1, 2 and 4), cluster 3 was characterized by the use of ceftriaxone/cloxacillin as a second-line antibiotics and the use of off-protocol antibiotics for the third line. Interestingly, cluster 3 had a higher odd of RD compared to both cluster 1 and 2. The severity of the disease impacts patient management as it trickles urgent use of broader spectrum antibiotics. As ceftriaxone broad spectrum focuses on gram-negative bacteria, clinicians decided to extend the spectrum by adding cloxacillin for gram-positive bacteria. When considering the survival probability, no differences were found based on antibiotic cluster assignment. The small sample size and the low number of patients in clusters 2, 3 and 4 may have led to this result. At the same time, it could also be related to the fact that prompt switch to a different line of antibiotics or the use of broader-spectrum antibiotics may have offset more severe infections or to the fact that some newborns may have died before switching antibiotic lines.

Cluster analysis also identified the frequency of antibiotic lines used, estimating the need to switch between lines due to ineffectiveness related to sepsis severity or antibiotic-resistant pathogens. In one out of four newborns of these cases (Cluster 2, 3 and 4), the first line antibiotic therapy with ampicillin plus gentamicin was not sufficient, in line with an increase in resistance to WHO-suggested first line therapy. The 5 common groups of Gram-negative bacteria (E. Coli, Klebsiella spp, Enterobacter spp, Pseudomonas spp, Acinetobacter), the main cause of neonatal sepsis in LMIC, are resistant to ampicillin and gentamicin in 40–100% of cases, depending to the bacteria involved [21–23].

The second line including a third-generation cephalosporin (i.e. ceftriaxone) required additional antibiotics in 12.3% of cases, leading to the emergence of clusters 3 and 4 in the present study. A review and meta-analysis in sub-Saharan Africa showed Gram-negative resistance to ceftriaxone ranging between 33% and 49% [23] while studies in Ethiopia reported higher resistance to cefotaxime (61.1-95.1%) [22, 24, 25]. Gram-positive bacteria showed resistance rates to third generation cephalosporin ranging from 50 to 95% [21–25], while resistance to fluoroquinolone and piperacillin/tazobactam is still low, ranging from 7 to 37% [25]. Yet, the number of multidrug resistant pathogens is increasing in Ethiopia [24, 25] and the above-mentioned alternative antibiotics are often unavailable and expensive, making the resistance to common antibiotics a very concerning issue [23].

Compared with high-income countries, the incidence of neonatal sepsis has been reported to be 40 times higher in LMICs [26]. In the present study, prevalence of neonatal sepsis in the overall patients admitted to the SLCH NICU (42.8%) was in line with recent Ethiopian data, showing a range from 33.6 to 40.7% [23, 25]. It should be noted that this prevalence was based on the diagnosis of probable neonatal sepsis based solely on clinical and laboratory findings. Confirmed sepsis, requiring a positive blood culture, was estimated in literature as one third of all probable neonatal sepsis [24, 28].

In our study, EOS had a prevalence three times higher than LOS. A study in Nepal showed a similar prevalence of EOS (71.2%) when considering clinically suspected neonatal sepsis. Higher EOS prevalence (between 84.1% and 90.2%) was reported in two studies conducted in different areas of Ethiopia [25, 27]. LOS were significantly more frequent in infants born at home and in health centers. This may be due to newborns receiving better care in hospitals as compared to home and health center births, where there is no trained or non- specialized staff and less equipment. In the study sample, EOS was associated with chorioamnionitis and PROM, two well-known risk factors for EOS development [18, 28].

Mortality rate was higher for infants with EOS (16.4%) compared to those with LOS (4.1%). A similar case fatality ratio was reported in literature for EOS (18%) and it was higher when compared to both community and hospital acquired LOS [29]. When evaluating the death and RD odds ratio in the study sample, it was higher as weight at admission decreased. Prematurity and low birth weight are well-known factors associated with the higher sepsis mortality and development of respiratory complications [30]. A neonatal mortality rate of 27 deaths per 1000 live births has been reported in sub-Saharan Africa for neonatal sepsis alone [31]. The achievement of the reduction of neonatal mortality in all countries to less than 12 deaths per 1,000 live births by 2030, envisioned by the Sustainable Development Goals, is jeopardized by the still high mortality from neonatal sepsis [32].

The main limitation of the study is the retrospective design and the small sample size of patients with neonatal sepsis and the small number of newborns assigned to clusters 2, 3 and 4. Cluster analysis, as an unsupervised learning technique, works best with large numbers. In addition, the small sample size did not allow the cluster to be used as a time-varying covariate making survival analysis to be interpreted with caution in the first 6 days since infants assigned to cluster 1 might have died before they had a chance to switch antibiotics and thus be assigned to another cluster. Nevertheless, to the best of our knowledge, this is the first study that seeks to apply an indirect technique to study antibiotic stewardship in settings where disease severity, patient complexity and lack of microbiological laboratory capacity leads to piling up of antibiotic prescriptions. Second, the lack of blood culture and pathogen isolation has prevented the diagnosis of confirmed neonatal sepsis and characterization of the antibiotic resistance profile. In addition, because of the lack of more specific blood tests (e.g., blood gas analysis) and blood cultures, some of the infants diagnosed with neonatal sepsis, based on symptoms and white blood cells, may actually have had perinatal asphyxia. Moreover, respiratory distress was collected as binary data; this prevented the application of a competing risk analysis. Finally, the study was conducted in a referral hospital and therefore involved a population of newborns that may have had more severe characteristics than those in peripheral health centers.

## Conclusions

The present proof of concept study used cluster analysis to depict the challenges of antibiotic management in a LMIC NICU. First-line antibiotics recommended by the WHO were not sufficient in one quarter of probable sepsis cases. Second-line treatment with third-generation cephalosporins was also insufficient in one in ten patients. The use of a machine learning technique allowed disentangling a patient group that received a different treatment than protocolized and more frequently evolved in RD. The use of different statistical methodologies should be encouraged to collect data where laboratory tests for sepsis typing and bacteria identification are not available so as to expand data pools from low resourced settings.

### Electronic supplementary material

Below is the link to the electronic supplementary material.

Supplementary Material 1

## Data Availability

The datasets generated and/or analysed during the current study are available from the corresponding author on reasonable request.
