# Supplementary material for: Exploring the use of cluster analysis to assess antibiotic stewardship in critically-ill neonates in a low resource setting
Source: Antimicrob Resist Infect Control. 2023 Oct 31;12:119. doi: 10.1186/s13756-023-01325-w (PMC10617092; doi:10.1186/s13756-023-01325-w)
Supplement: Supplementary file 1 — Supplementary Material 1 [file 13756_2023_1325_MOESM1_ESM.docx]

**SUPPLEMENTARY MATERIAL**

Figure S1 – Silhouette coefficient based on the number of clusters fitted over the Gower distance estimated on type of antibiotics used as the first, second, or third line, respectively, and the length in days of each antibiotic line.


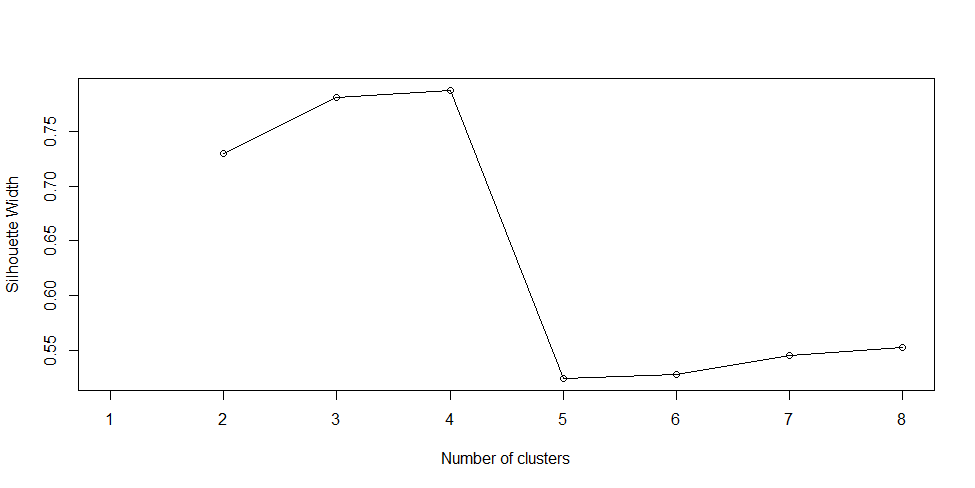


Table S1 - Demographic, epidemiological, and clinical characteristics of newborns with probable neonatal sepsis, distinguished by the cluster of antibiotic management.

|  | **cluster 1 (n=145)** | **cluster 2 (n=26)** | **cluster 3 (n=15)** | **cluster 4 (n=9)** | **p-Value** | **Overall (n=195)** |
| --- | --- | --- | --- | --- | --- | --- |
| **Sex** |  |  |  |  | 0,693 |  |
| Female | 59 (40,7%) | 11 (42,3%) | 8 (53,3%) | 5 (55,6%) |  | 83 (42,6%) |
| Male | 86 (59,3%) | 15 (57,7%) | 7 (46,7%) | 4 (44,4%) |  | 112 (57,4%) |
| **Age (day)** |  |  |  |  | 0,025 |  |
| median (IQR) | 2,0 (1,0-8,0) | 1,0 (1,0-2,0) | 1,0 (1,0-1,0) | 1,0 (1,0-1,0) |  | 1.0 (1.0-5.5) |
| **Weight at admission (g)** |  |  |  |  | 0,432 |  |
| median (IQR) | 2865 (2400-3250) | 2950 (2372-3338) | 3000 (1850-3550) | 2600 (2250-2800) |  | 2900 (2315-3300) |
| **Premature rupture of membranes (N/A=2)** |  |  |  |  | 0,618 |  |
|  | 29 (20,0%) | 8 (30,8%) | 3 (20,0%) | 1 (11,1%) |  | 41 (21,0%) |
| **Chorioamnionitis (N/A=2)** |  |  |  |  | 0,012 |  |
|  | 12 (8,3%) | 1 (3,8%) | 2 (13,3%) | 4 (44,4%) |  | 19 (9.7%) |
| **Eclampsia (N/A=2)** |  |  |  |  | 0,998 |  |
|  | 3 (2,1%) | 0 (0,0%) | 0 (0,0%) | 0 (0,0%) |  | 3 (1,5%) |
| **Breath at birth (N/A=2)** |  |  |  |  | 0,011 |  |
|  | 120 (82,8%) | 20 (76,9%) | 7 (46,7%) | 8 (88,9%) |  | 155 (79,5%) |
| **Positive pressure ventilation at birth (N/A=2)** |  |  |  |  | 0,039 |  |
|  | 24 (16,6%) | 7 (26,9%) | 7 (46,7%) | 1 (11,1%) |  | 39 (20,0%) |
| **Oxygen given at birth (N/A=2)** |  |  |  |  | 0,538 |  |
|  | 33 (22,8%) | 6 (23,1%) | 6 (40,0%) | 2 (22,2%) |  | 47 (24,1%) |
| **Sepsis type** |  |  |  |  | 0,274 |  |
| EONS | 103 (71,0%) | 22 (84,6%) | 13 (86,7%) | 8 (88,9%) |  | 146 (74,9%) |
| LONS | 42 (29,0%) | 4 (15,4%) | 2 (13,3%) | 1 (11,1%) |  | 49 (25,1%) |
| **Respiratory distress syndrome** |  |  |  |  | 0,001 |  |
|  | 59 (40,7%) | 13 (50,0%) | 13 (86,7%) | 7 (77,8%) |  | 92 (47,2%) |
| **Outcome at discharge** |  |  |  |  | 0,181 |  |
| alive | 125 (86,2%) | 25 (96,2%) | 11 (73,3%) | 8 (88,9%) |  | 169 (86,7%) |
| dead | 20 (13,8%) | 1 (3,8%) | 4 (26,7%) | 1 (11,1%) |  | 26 (13,3%) |
| **Length of stay** |  |  |  |  | <0,001 |  |
| median (IQR) | 7,0 (6,0-8,0) | 12,5 (10,0-15,8) | 11,0 (8,0-23,5) | 17,0 (14,0-18,0) |  |  |
| **Apgar 1^st^ minute** |  |  |  |  | 0.430 |  |
| median (IQR) | 6,0 (4,0-8,0) | 6,0 (3,3-7,8) | 3,0 (2,0-6,5) | 6,0 (5,0-7,0) |  | 6.0 (3.0-8.0) |
| **Apgar 5^th^ minute** |  |  |  |  | 0,264 |  |
| median (IQR) | 7,5 (6,0-9,0) | 7,0 (5,3-9,0) | 5,0 (4,5-7,5) | 7,0 (6,5-7,5) |  | 7.0 (6.0-9.0) |

*Table S2 – Output of the cluster analysis through k-medoids algorithm. The table shows the number of individuals assigned at each cluster distinguished by the type of antibiotic combination in every antibiotic line and the median duration of every antibiotic line with the interquartile range.*

|  | **Type** | **Number (%) of neonates**  **(total=195)** | **Duration of antibiotic treatment in days** |
| --- | --- | --- | --- |
| **Cluster 1** |  |  |  |
| First line | AMP + GEN | 145 (74.4%) | 6.0 [5.0-7.0] |
| Second line | none | 145 (74.4%) | N/A |
| Third line | none | 145 (74.4%) | N/A |
| **Cluster 2** |  |  |  |
| First line | AMP + GEN | 26 (13,3%) | 6.5 [4.1-7.0] |
| Second line | CTX | 26 (13,3%) | 7 [5.3-7.0] |
| Third line | none | 25 (12,8%) | N/A |
|  | VAN | 1 (0,5%) | 8.0 [8.0-8.0] |
| **Cluster 3** |  |  |  |
| First line | AMP + GEN | 15 (7,7%) | 6.0 [4.5-7.5] |
| Second line | CTX+CLOXA | 12 (6,2%) | 5.0 [3.0-6.3] |
|  | CTX+CLOXA+AZM | 3 (1,5%) |  |
| Third line | CPFX | 1 (0,5%) | 7.5 [7.0-9.7] |
|  | CPFX+VAN | 2 (1,0%) |  |
|  | CPFX+VAN+TZP | 1 (0,5%) |  |
|  | PCN | 1 (0,5%) |  |
|  | none | 10 (5,1%) | N/A |
| **Cluster 4** |  |  |  |
| First line | AMP + GEN | 9 (4,6%) | 5.5 [5.0-7.0] |
| Second line | CTX | 9 (4,6%) | 4.0 [4.0-4.0] |
| Third line | CPFX+CLOXA | 7 (3,6%) | 9 [7.0-9.5] |
|  | CPFX+VAN | 1 (0,5%) |  |
|  | CPFX | 1 (0,5%) |  |
| **AMP=Ampicillin, GEN=Gentamicin, CTX=Ceftriaxone, VAN=Vancomycin, CLOXA=Cloxacillin, AZM=Azithromycin, CPFX=Ciprofloxacin, TZP= Piperacillin /Tazobactam, PCN=Penicillin.* | | | |
